# Influence of Immunogenetic Biomarkers in the Clinical Outcome of HTLV-1 Infected Persons

**DOI:** 10.3390/v11110974

**Published:** 2019-10-23

**Authors:** Antonio Carlos Rosário Vallinoto, Izaura Cayres-Vallinoto, Maria Alice Freitas Queiroz, Marluísa de Oliveira Guimarães Ishak, Ricardo Ishak

**Affiliations:** Laboratório de Virologia, Instituto de Ciências Biológica, Universidade Federal do Pará, Belém 66.075-110, Brazil; ivallinoto@ufpa.br (I.C.-V.); alicefarma@hotmail.com (M.A.F.Q.); marluisa.malu@gmail.com (M.d.O.G.I.); rishak@ufpa.br (R.I.)

**Keywords:** HTLV-1, SNPs, immunogenetic background, biomarkers, retroviridae

## Abstract

Human T-lymphotropic virus 1, a member of the *Retroviridae* family, causes a neglected, silent, persistent infection affecting circa 5 to 10 million people around the world, with biology, immune pathology, clinical diseases, epidemiology, and laboratory issues still unsolved. Most of the infected subjects are asymptomatic, but severe clinical disorders appear as a neurodegenerative disease (HTLV-1 associated myelopathy—HAM) or a lymphoprolipherative disorder (Adult T Leukemia/Lymphoma—ATLL) and in other target organs of the human body. HTLV-1 infections are frequently asymptomatic, but there is a large spectrum of diseases that have been described along the years. The mechanisms by which the virus interacts with the host, the different modes of response of the host to the infection, and the immunogenic characteristics of the host are some of the interesting and unanswered questions that may direct the outcome of the disease. The most relevant published results dealing with the genetic variations of the host, the immune response to HTLV-1 infection, and the outcome of the infection are presented herein, including Human Leucocyte Antigen (HLA), Killer Immunoglobulin-like Receptors (KIR), interleukin 6, 10, 28, Fas and Fas ligand, IFN-gamma, TNF-A, and Mannose-binding lectin. In summary, there are still several unmet research needs in the field of useful biomarkers on HTLV-1 pathogenesis.

## 1. Introduction

Human T-lymphotropic virus 1 (HTLV-1) is a *Deltaretrovirus*, endemic in Japan, Caribbean, South America, Sub-Saharan Africa, and Melanesia [1,2,3].

HTLV-1 is associated with several different types of diseases. Adult T-cell leukemia/lymphoma (ATLL) develops in up to 5% of infected people, and sometimes a chronic neurodegenerative inflammatory disease called HTLV-1-associated myelopathy (HAM) develops. Although a high percentage of people remain asymptomatic during their lifetime [4], a great number of infected people develop a large range of neurological diseases [5]. Other opportunistic-like infections, such as Strongyloidiasis, Norwegian scabies, infective dermatitis, as well as autoimmune syndromes, including rheumatoid arthritis and Sjögren’s syndrome, have also been described among HTLV-1 carriers. In these patients, the clinical conditions appear to be the result of the interaction between the virus and the host susceptibility, leading to the abnormal functioning of immuno-modulatory mechanisms, cell proliferation, and inflammation [3,4,6].

HAM is a chronic progressive demyelinating disease that affects the spinal cord and the white matter of the brain, leading to a severe clinical syndrome due to the motor limitations that affect the lower limbs associated with the autonomic dysfunction. The clinical picture begins and evolves insidiously, being difficult to establish when the first symptoms appeared. The main signs and symptoms of HAM may include gait disturbance, weakness, and stiffness of the lower limbs. The first manifestations of the disease occur in the fourth decade of life and there is a 2:1 woman/man ratio [7,8]. The incubation period differs from patient to patient and seems to be shorter in the cases where the virus was acquired through blood transfusion [6,9,10,11,12].

Two important aspects of HAM development have been reported, including the efficiency of the cellular immune response and the rate of spontaneous expression of the viral antigen, due to the numerous immunological alterations, mediated mainly by the expression of Tax (regulatory protein of the virus) in the peripheral blood and in the cerebrospinal fluid of patients with HAM [13,14].

In order to control HTLV-1 replication and the proliferation of infected cells, CD8^+^ T lymphocytes mediate the main defense strategy of the host immune system [4,15]. HTLV-1 infects mainly CD4^+^ T cells, which are the central regulators of the acquired immune response, deregulating the host immune system [16]. To establish persistent infection, HTLV-1 deregulates CD4^+^ T cells, sometimes leading to ATLL [17] or to chronic inflammatory diseases, such as HAM and uveitis [7,18,19,20,21].

The absence of symptoms in most of the infected people raises the question of the multiple etiological roles of the virus-associated diseases and some of the interesting questions still unanswered includes: does the immunogenic characteristics of the host contribute to the upsurge of symptoms associated with HTLV-1 infection? The present manuscript presents the most relevant published results from several laboratories, including ours over the years regarding the associations between the genetic variations of the host related to the immune response and HTLV-1 infection, particularly those showing the close relationship between HTLV-1 infection and single nucleotide polymorphisms in genes of the immune system.

## 2. HTLV-1 Infected Subjects’ Immunogenetic Profile

The interaction between HTLV-1 and the host immune response appears to play an important role in the outcome of HTLV-1-induced diseases [22,23], since anti-HTLV specific CD8^+^ T lymphocytes play an important role in limiting viral replication in vivo reducing the proviral load and the number of infected CD4^+^ T lymphocyte clones [6]. Thus, several investigations have focused on host factors and virus-host interactions that contribute to the pathogenesis of HTLV-1 infection. Studies developed among African and Japanese descendants suggested that HTLV-1 infection, as well as related diseases, could be determined according to the immunogenetic background of the host [24,25].

### 2.1. Human Leukocyte Antigen (HLA) and KIR Genes

Human leukocyte antigen (HLA) is of utmost importance in inducing the immune response against HTLV-1 and is a critical factor affecting the proviral load of the virus ([26,27]; Table 1). The interaction with the immature free form of the HLA class I heavy chain molecule, the viral protein p12^I^ facilitates the replication of HTLV-1 and allows the virus to escape the host immune response, preventing the presentation of viral proteins by HLA class I molecules to cytotoxic T lymphocytes; p12^I^ inhibits the expression of HLA-1 molecules on the surface of infected cells [28].

Specific HLA alleles have been related to the protection of HAM development, while others appear to increase the risk for the development of ATLL [26]. HLA-DRB1 haplotypes that are usually correlated with lowered immune response were associated with the presence of ATLL, whereas those correlated with high immune response were associated with HAM [24]. Further studies have also confirmed the association between HLA-DRB1*01 and the increased risk of HAM in HTLV-1 infected individuals [33,35,39]. However, Nishimura et al. [30] did not find any association between HLA-DRB1, ATLL, and HAM.

There are different mechanisms for each of the disease outcome. The HLA-DRB1*01 allele, like certain types of HLA class II, seems to present peptides more efficiently to virus-specific CD4^+^ T cells in patients with HAM, resulting in a higher viral burden and increased inflammatory response, which in turn indirectly intensifies the damage of the central nervous system [47].

In Japan, HLA-A*02 was associated with protection against the development of HAM [26,27]. A strong antiviral cytotoxic T response is initiated by individuals with this type of HLA, because of its high binding affinity for Tax peptides (residues 11–19), resulting in the elimination of HTLV-1 infected T cells and the subsequent reduction of the proviral load, leading to the protection to HAM [26]. However, the frequency of HLA-A*02 allele was not significantly higher among asymptomatic HTLV-1 carriers as compared to HAM patients [32].

In addition, patients with ATLL also presented this allele. Nucleotide sequence analysis indicated that these patients have variations in the tax gene, which generate a stop codon in the half of exon 5 or generate an amino acid change in the critical Tax epitope, which leads to the evasion of the immune response in HLA-A*02 with ATLL [31]. Individuals expressing HLA class I related to the predisposition to the development of ATLL (HLA-A*26, HLA-B*402, HLA-B406 e, HLA-B*4801), exhibit modified peptide binding sites that are unable to recognize different HTLV-1 Tax protein peptides. They are unable to generate an efficient population of Tax-specific cytotoxic T lymphocytes [32]. But in Iran, Tarokhian et al. [42] did not observe any evidence of susceptibility or protection role to HLA-A*02, HLA-A*24, HLA-Cw*08, and HLA-B*5401 alleles in symptomatic and asymptomatic HTLV-1 infected people. Taghaddosi et al. [40] could not find any association between HLA-I alleles (HLA-A*02, HLA-A*24, HLA-Cw*08) and the risk for HAM. In Japan, Jeffrey et al. [27] also associated HLA-B*5401 allele with higher proviral load and susceptibility to HAM. In Jamaica, Goedert et al. [34] reported a low risk for ATLL in HTLV-1 infected people carrying alleles A*03 and DQB1*0501, but a higher risk for those carrying B*15 and B*53 alleles.

At this point, it is relevant to stress that some HLA alleles that seem to be protective in one population may not have a similar effect in a different population [27,32,35,36,42]. This may be a consequence of the complex interaction of factors, including pathogen variation, host genetic heterogeneity, and allelic frequency in the studied population [40]. It is relevant to stress that the differences found among studies may be attributed to variables that are not the same for different geographical areas, including sample size and ethnic background of the investigated populations.

Different types of HLA class I were associated with the HBZ protein of HTLV-1, a regulatory protein which promotes T lymphocyte proliferation and is correlated with disease severity [48]. Although HLA class I alleles, A*0201 and Cw*0801, were associated with increased binding strength to HBZ, other types of HLA also conferred a protective effect against disease development and increased HTLV-1 proviral load [36]. In Iran, Rafatpanah et al. [35] reported the presence of HLA-Cw*08 allele in high frequency among HAM patients and associated with low CD8^+^ T lymphocyte immune response. This was in clear contrast to results observed by Tarokhian et al. [42] that, investigating the same Mashhad population, did not find association of the same allele with HAM. It is possible that the differences could be attributed to the distinct PCR methods used to define the presence of the alleles and genotypes.

Non-classical HLA Class I, for instance, HLA-G, was associated with HTLV-1 infection. The 14 bp polymorphism (deletion/insertion) in the 3’-UTR region (HLA-G 3’-UTR) was not linked to diseases associated to HTLV-1, but it could be a risk factor for susceptibility to HTLV-1 infection [38]. But for further conclusions, the association with susceptibility to HTLV infection should take into account the risk of exposition. The 3’-UTR region of HLA-G exhibits great genetic variability, which can in post-transcriptional level modify its production and contribute to the progression of HTLV-1 infection, the magnitude of HTLV-1 proviral load, and the outcome to HAM [41].

Another important HLA class I activity is its binding to killer immunoglobulin-like receptors (KIR) expressed on the surface of NK cells. The HLA-KIR interaction may promote the inhibition or the activation of NK cell functions [49]. Genes that code for KIR present a large genetic variability, resulting in different types of receptors [50].

The initial approach to infer the influence of KIR on HTLV-1 infection was possible action of specific KIR genes and HLA-Cw groups in the development of HAM. Although there was a trend of association of genes with neuropathology and proviral load, in the final step, no significant associations were found between KIR genes and HLA-Cw groups in people with a clinical diagnosis of HAM [37].

KIR2DL2 binds to HLA-C molecules with different levels of affinities, and a high affinity binding to the C1 group containing asparagine at residue 80 has been described, as well as links with a low affinity to HLA-C2 molecules when there is a lysine at position 80 [51]. In HTLV-1 infection, KIR2DL2 gene can both enhance protection and impair HLA class I-mediated immunity against HTLV-1 infection. KIR2DL2 was associated with HLA-C*08 with a low proviral load in asymptomatic patients and HLA-B*54 with a high proviral load in HAM patients. The activity of KIR2DL2 appears to enhance immunity against HTLV-1 through the activation of two cell types, NK cells and CD8^+^ T lymphocytes, both of which may exert better control in the development of clinical disease [45].

The evaluation of the protective effect of HLA-B*57 on different types of KIR (KIR2DL1^+^2^+^, KIR2DL2^+^C1^+^, KIR2DL2^+^C2^+^, KIR2DL3^+^C1^+^, KIR2DL1^-^ or KIR2DL1^+^C2^-^, KIR2DL3^-^ or KIR2DL3^+^C) showed that they all enhance HLA class I associations in HTLV-1 infection. These findings may be related to increased survival of CD8^+^ T cells in the presence of functional KIRs [44].

In contrast, other studies found no association between polymorphisms in HLA and KIR alleles with HTLV-1 infection. Although KIR3DS1 expression has been associated with host response in HIV infection, KIR3DS1-mediated regulation on HTLV-1 infection does not occur or is ineffective [46]. Likewise, other polymorphisms in the HLA-C and KIR alleles were also not associated with the risk of progressing to HAM [43].

Although the importance of HLA and KIR in the anti-viral immune response is recognized, there is a broader lack of studies involving different ethnic groups that seek to identify the possible impact of polymorphisms in these genes on HTLV-1 infection and associated diseases. However, it is worth mentioning that the disagreement found among different studies can be mainly due to the small sample sizes used, the ethnic background, and the population stratification effect.

### 2.2. Cytokines Genes

Several cytokines may influence both the host immune response and HTLV-1 proviral load levels and may be directly involved in the genesis of different pathologies [22]. In fact, there is an association between uveitis and the presence of polymorphism in tumor necrosis factor gene [29]. Polymorphism in lymphotoxin-α (LT-α) and TNF-type 2 receptor genes were associated with a high proviral load, suggesting positive influence on viral replication in HAM patients [30].

The factors that determine the genesis of HAM, as well as the differences in proviral load between symptomatic and asymptomatic carriers of HTLV-1, are still not well understood. However, in view of the previous evidence, the importance of the host’s genetic profile affecting the immune response generated against the virus became a relevant line of research in the pathogenesis of the virus ([22]; Table 2).

Neco et al. [53] investigated the presence of *IL17A* polymorphisms among HTLV-1 infected subjects and did not find an association of allele and genotype frequencies with the status of symptomatic and asymptomatic carriers. The absence of statistical significance, however, could be attributed to the small sample size, which is a difficult task to overcome when dealing with HTLV-1 infection.

IFN-γ, an important proinflammatory cytokine, is commonly associated with the pathogenesis of HAM [75,76,77] and among asymptomatic individuals, the polymorphic allele for the *IFNG* +874A/T polymorphism was associated with higher levels of IFN-γ and proviral load, and a more constant follow-up was suggested for these patients to detect possible infection-related symptoms [54].

The investigation of IL-18 (−137C/G and −607A/C) and IFN-γ (+874T/A) gene polymorphisms among asymptomatic and symptomatic HTLV-1-infected subjects, and in control individuals, showed that *IFNG* +874A/T genotype was associated with a higher proviral load than +874AA genotype [55]. The frequency of the *IL18* −607CC genotype was significantly lower in HTLV-1-infected individuals compared to uninfected people. On the other hand, the *IL18* −607AC genotype was significantly more frequent among HTLV-1-infected subjects. Haplotype analysis showed the *IL18* −137G/−607A haplotype was more frequent in the infected group compared to the uninfected control group, and the *IL18* −137C/−607C haplotype was increased in the control group compared to the others, suggesting that the presence of *IL18* −607AC genotype and *IL18* −137G/−607A haplotype could be a possible biomarker for increasing the risk factor for infection, in opposition to the presence of *IL18* −607CC genotype and −137C/−607C haplotype, that appear to confer protection against HTLV-1.

TNF-α is another significant cytokine in the development of symptoms induced by HTLV-1 infection. Higher TNF-α levels were associated with the *TNFA* −857C/T polymorphism, related to the high transcriptional activity of the gene. The polymorphic genotype was associated with a 2-fold increase in the risk of developing ATLL [57]. An interaction between *TNFA* 1031T/C and HTLV-1 was also reported for atherosclerosis-related disease [58].

IL-10 can influence HTLV-1 infection through its immunoregulatory functions and inhibit the differentiation process of specific cytotoxic T cells. HTLV-1 infected individuals with low levels of IL-10 may allow inflammatory mechanisms that lead to HAM [61]. The wild-type allele of the *IL10* −592A/C polymorphism was associated with HTLV-1 transcriptional activity mediated by the Tax protein, influencing both HTLV-1 proviral load and a 2-fold reduction on the chances of developing HAM [59]. According to Schor et al. [56] the *IL10 −592AA* genotype is associated with higher proviral load. However, the investigation of this polymorphism, together with others located in the *IL10* gene, showed that *IL10* −819*C/T and −592*C/A polymorphisms may contribute to susceptibility to infection by HTLV-1, although it does not appear to be related to the protection against HAM development [61]. In Brazil, Braz et al. [62] reported a protective effect of allele *A of IL-10 rs1800896 polymorphism to neurological symptoms (over-active bladder) among HTLV-1 patients, suggesting a regulatory role to IL-10.

IL-6 is an important pro-inflammatory cytokine of acute phase and in the defense against infectious agents [78]. Changes in the *IL6* promoter region were associated with the development of HAM [52]. However, not all genetic variations located in the *IL6* gene promoter region are related to HTLV-1 infection and disease development. Among several investigated SNPs (positions −174, −572, −597 and −634), *IL6* −634G/C allele was described to be a significant predictor for the progression to HAM [60].

Susceptibility to HTLV-1 infection in children was linked to haplotypes *IL6* −660G/−635C/−236G and *IL10* −6653C/−1116G, independently of maternal provirus load and duration of breast-feeding, suggesting that host’s genetic variations, for both proinflammatory and anti-inflammatory cytokines genes, might be influencing the susceptibility to infection. The relationship of these cytokines to HTLV-1 infection is evidenced in the different activities of the immune response to infection. IL-6 induces the production of IL-2, a cytokine crucial for the activation, growth, and differentiation of cytotoxic T lymphocytes, important in establishing the inflammatory process of HAM. However, IL-10 may inhibit these activities. Therefore, polymorphisms in one of these genes may favor infection and the development of diseases related to infection [79]

In contrast, Schor et al. [56], in a case-control study, reported the absence of association between single nucleotide polymorphisms of pro- and anti-inflammatory cytokines (*TNFA* −308G/A, *IL6* −174G/C, *IFNG* +874T/A, *TGFB*) and HAM patients from Brazil.

Interleukin 28B (Interferon-λ 3, IFNL3) plays an important role in immune defense against viruses, by inducing an “antiviral state” by intracellular signaling and activation of antiviral host factors in susceptible cells. The role of IL-28B in the context of infectious diseases may be influenced mainly by polymorphisms in the gene that encodes the cytokine [80].

There are few studies that seek an association of polymorphisms in the IL-28B gene with HTLV-1 infection, but they showed that polymorphisms of this gene are linked with the progression of HTLV-1 infection [63] and that they play an important role in the HAM pathogenesis [64,66]. The TT genotype of the rs8099917 polymorphism was related to a high expression of the *IL28B* gene [63]. However, high levels of IL-28B were associated with the development of ATLL and HAM [81]. High *IL28B* expression levels are mainly associated with high levels of Tax protein [82] as a consequence of the disorganized proliferation of CD4^+^ T lymphocytes leading to marked development of the inflammatory process characteristics, mainly HAM. Another study showed that patients with the CT/TT genotype of rs12979860 polymorphism have a greater proviral load than those with the CC genotype, thus suggesting that the presence of *T allele might be associated with the pathogenesis of infection and disease outcome contributing to the progression to HAM [64].

However, Sanabani et al. [65] failed to find any association of polymorphism rs12979869 of IL28B gene with HTLV infection and its progression to HAM. Though, an association between the SNPs rs8099917 and rs12979618 with distinct host immune response against HTLV-1 has led to a suggestion that *IL28B* polymorphisms should be investigated for all HTLV-1-infected subjects in order to monitor their risk for disease development [66].

We investigated the association of the single nucleotide polymorphisms (rs8099917, rs12979860, and rs8103142) in the *IL28B* gene and the development of HTLV-associated arthropathy (HAA), but no differences were observed in the allele and genotype frequencies between symptomatic and asymptomatic HTLV-1 infected people [67]. Seven haplotypes were detected, being the haplotype CCT, the most common among the HTLV-infected subjects, and haplotype TTG, present only in the HAA group. Individuals with HAA and genotype TT (rs8099917) showed a larger CD8^+^ T lymphocyte count and higher proviral load as compared to asymptomatic. Furthermore, high TNF-α and IFN-γ levels serum were observed in HAA patients carrying genotypes CC (rs12979860) and TT (rs8103142). On the other hand, HAA patients with genotype CT/TT (rs12979860) exhibited high IL-10 levels. These results suggest that haplotypes CCT and TTG might be associated with susceptibility to HTLV infection and progression to HAA, respectively.

Considering the role of cytokines in the signaling and activation of mediators of innate and adaptive immune response, it seems important to broaden the analysis of SNPs in genes of pro and anti-inflammatory cytokines in the search to identify possible haplotypes associated with the regulatory and activating functions of the immune response.

### 2.3. Apoptosis Genes

Apoptosis mechanisms are highly complex and sophisticated, involving a cascade of energy-dependent molecular events [83]. The process of apoptosis can be initiated by external stimuli, through the activation of specific receptors present on the cell surface, called death receptors, or by intracellular stress, characterizing the main pathways of activation, extrinsic or via death receptor and intrinsic or mitochondrial, respectively [84,85].

Both pathways culminate in the activation of proteases known as caspases, which are responsible for a cascade of events that lead to the appearance of cellular changes characteristic of all cells in apoptosis, and the action of these proteases culminates in a common effector path independent of stimulant [86,87].

As death domain-containing member of the TNFR (tumor necrosis factor receptor) superfamily, the Fas (TNFRSF6/CD95/APO-1) and FasL (TNFSF6/CD95LG) molecules are involved in physiological regulation of programmed cell death [88,89], being implicated in the pathogenesis of various malignancies and diseases of the immune system.

A single nucleotide polymorphism (SNP) at position −670 (*FAS* −670 A > G; rs 1800682) was identified in the *FAS* gene promoter region [90,91]. On the other hand, three polymorphisms have been reported within the *FASLG* gene, two of them include a T deletion in position −169 of intron 3 (*FASLG* IVS3nt −169 T > ΔT; ni) and an A to G substitution at nucleotide position −124 of intron 2 (*FASLG* INV2nt −124 A > G; rs 5030772) [92,93,94].

The gene location of *FAS* −670A/G SNP seems to favor the binding to activator of transcription (STAT1), leading to up and down regulation of the *Fas* gene expression. Farre et al. [68] showed that this polymorphism is significantly associated to the susceptibility to HTLV-1, clinical manifestation, and survival of HTLV-1 patients presenting ATLL. However, it remains unclear how this polymorphism could influence the susceptibility to infection.

FAS levels were linked positively with lymphocyte activation among HAM patients, but the increased lymphocyte FAS expression was associated with: (i) decreased apoptosis, (ii) high lymphoproliferation in cell culture, and (iii) a strong increase in cellular Fas expression upon HAM progression [95].

Recently, *FAS* −670 AA genotype was associated with high proviral load as compared to *FAS* −670 GG in HAM patients [70]. This result is in agreement with our study, where the genotype *FAS* −670AA was more frequent among HAM patients as compared to the asymptomatic individuals [69], suggesting that individuals carrying this genotype have a greater chance and a higher susceptibility to progress to overt clinical disease. Probably, it is due to allele *A being associated with high apoptosis activation, as reported by Khouri et al. [71], which lead to death of the infected Schwann cells followed by demyelination, as observed in the peripheral nerves of these rats [96]. Furthermore, in a recent report, we show, for the first time, a family cluster of diseased people infected by HTLV-1 across three generations associated with *FAS* −670A/G polymorphism [97]. In this study, the allele *A was present among three symptomatic HTLV-1 infected sisters and absent among the others asymptomatic HTLV-1 infected family members. Thus, *FAS* −670A/G polymorphism seems to be a reliable biomarker associated to HTLV infection and further study involving larger numbers of other families should be conducted to confirm the association observed until now.

Polymorphisms in other genes related to apoptosis (*GSTM1*, *GSTT1*, and *BCL2*) were investigated, but no association with ATLL was observed in Japan [57].

### 2.4. Mannose Binding Lectin Gene

Mannose binding lectin (MBL) is an acute phase serum protein that recognizes structures of carbohydrates arranged in a particular geometry, found on the surface of microorganisms. MBL binds to serine proteases (MASPs) forming the complex, which promotes activation of the complement cascade via the lectin route [98].

As previously mentioned, polymorphism in the major histocompatibility complex has shown a possible association with HTLV-1 infection and related pathologies [22]. However, other genetic markers may be involved in this process [99]. As an example, we can cite the polymorphism in the mannose binding lectin gene that probably seems to influence the immune response to HTLV-1 and, consequently, to the proviral load of infected individuals [72]. However, there are few studies in this area, and it needs further clarification over the polymorphism in this gene in relation to HTLV-1 infection.

Our group investigated the association between MBL2 gene exon1 polymorphisms and the susceptibility to, as well as the outcome of, HTLV-1 and HTLV-2 infections [73].

Regarding HTLV-1/2, the frequencies of alleles MBL*A, MBL*B, and MBL*D were similar between seropositive individuals and healthy controls, but genotype differences were statistically significant; where the genotype BB, related to low MBL production, was 9-fold higher among HTLV-infected individuals as compared to controls, suggesting a possible association with susceptibility to HTLV infection. The same difference was observed in genotype distribution between HTLV-1 and HTLV-2 infections, where it was attributed to the higher number of HTLV-1-infected subjects investigated [73].

Subsequently, we analyzed the frequencies of SNPs at position −550 and −221 of the *MBL2* gene promoter [74]. No significant difference was reported between the HTLV-1/2 infected and control groups, but there was a significant difference at position −221. A higher prevalence of genotype LYLX, usually associated with medium and low MBL serum levels, was associated with the risk of HTLV-1/2 infection, though no association between proviral load and the promoter polymorphism had been observed. However, when the promoter and exon 1 mutations were matched, a significantly higher proviral load was detected in the HTLV-1/2 infected individuals carrying haplotypes correlated with low serum MBL levels.

Subsequently, it was demonstrated that combined genotypes associated with low or deficient production of MBL2 (LXA/LXA, HYA/O, LYA/O, LXA/O, or O/O) were significantly associated with susceptibility to HTLV-1/2, and more frequently in patients infected with HTLV-1/2 than in the controls. Moreover, the combination of genotypes with high protein production (HYA/HYA, HYA/LYA, HYA/LXA, or LYA/LXA) were significantly associated with protection against HTLV-1. These results showed that the deficiency of the components of innate immunity may play a role in susceptibility to HTLV-1 infection [100].

The investigation of the polymorphisms in the *MBL2* gene has shown its relevance in the anti-HTLV response, especially in association with the proviral load. However, the small number of published articles and small sample sizes emphasizes the need for a broader evaluation, taking into account the ethnic differences of populations infected by HTLV-1 in order to better understand the relationship of the *MBL2* gene with the infection.

## 3. Conclusions

HTLV-1 infections are commonly thought to be asymptomatic, but there is a large spectrum of diseases which are currently being described along the years showing that the infection, by itself, cannot be the only cause for the progression towards HTLV-1 associated diseases. So far, there are no clear explanations why some people present a full-blown disease like HAM or ATLL, others develop milder diseases with few symptoms, and some are clearly asymptomatic.

HTLV-1 is a genetically stable virus and individuals with the same virus strain have been reported with different clinical outcomes. There is strong evidence that HTLV-1 proviral load is a risk factor for disease progression. Proviral load is directly dependent on the strength of the host cytotoxic T lymphocyte response to HTLV-1, which in turn, is genetically associated with HLA class I type and cytokines signaling. The role of immunogenetic markers of the host is routinely investigated in our laboratory in order to find some explanation for the mechanism of the upsurge of symptoms in some point of the infection. Information regarding the action of polymorphisms and expression of the host genetic characteristics of IFN, TNF, Fas, FasL, IL-6, IL-8, IL-10, IL-28B, and MBL are clear indicators that the immunogenetic profile contributes to the appearance of symptoms as a consequence of HTLV-1 infection.

It is extremely relevant to comment that most of the inconsistent results observed from the same biomarker in different human populations could be due to the complexity of epigenetic factors, population stratification effect, the small sample size, ethnic background, covariates, consistency of biomarkers described, and replication in different populations, which can interfere in case-control association studies producing false association results.

It is important to stress that we are still trying to merge all the information of the few, but comprehensive, information so far generated by us and other laboratories to understand the synergism of the several biomarkers exploited. Recently, apart from this line of investigation, our laboratory has pointed into the direction to search for new biomarkers and found that the lowest expression of Annexin 1 gene and its serum levels act as a possible new biomarker associated to HAM clinical outcome [101].

In summary, there are still several unmet research needs in the field of useful biomarkers that could help to answer questions such as: who is more easily infected with HTLV-1? Who preferentially becomes sick following infection? Which outcome of clinical disease(s) is linked to which biomarker(s)? In order to fulfill these and other answers, there should be an effort to increase the number of studies in search for genetic biomarkers in different human populations, expanding both the biodiversity of the human groups and the number of subjects investigated in order to confirm or reject the suggestions presented herein. HAM, ATLL, and all the other clinical manifestations of HTLV-1 infection are severe diseases of the poor population of the world, which remains silent and neglected.

## Figures and Tables

**Table 1:** Main polymorphisms in HLA and KIR genes associated with HTLV-1 infection.

Gene	Alleles/Genotype	Country	Method	N	Disease	Association	Reference
**HLA**	A*26, B*61, and DR9	Japan	PCR-SSP	330	ATLL	Risk	[24]
	A*24 and Cw*01				ATLL	Protective	
	Cw*7, B*7 and DR1				HAM	Risk	
	B*61 and DRB1*0901	Japan	PCR-SSOPs	652	Uveitis	No	[29]
	A*02	Japan	PCR-SSP	433	HAM	Protective	[26]
	DRB1*0101				HAM	Susceptibility	
	A*02, Cw*08	Japan	PCR-SSP	435	HAM	Protective	[27]
	B*5401				HAM	Susceptibility	
	DRB1* 0405, DRB1*09	Japan	PCR-SSP	261	HAM	No	[30]
					ATLL	No	
	A*02	Japan	PCR-SSP	178	ATLL	Susceptibility	[31]
	*A26, Cw*08, B*4002, B*4006, and B*4801	Japan	PCR-SSP ARMS	599	ATLL HAM	Susceptibility No	[32]
	DRB1*0101	Iran	PCR-SSP	132	HAM	Susceptibility	[33]
		Japan	PCR-SSP	406	HAM	Susceptibility	
	A*02	Iran	PCR-SSP	132	HAM	No	
		Japan	PCR-SSP	406	HAM	No	
	DRB1*1503 and DQB1*0602	Jamaica	PCR-SSP PCR-SSO	195	HAM	Low risk	[34]
	A*03 and DQB1*0501				ATLL	Low risk	
	B*15 and B*53				ATLL	High risk	
	DRB1*01	Iran	PCR-SSP	142	HAM	Susceptibility	[35]
	Cw*08					Low CTL response	
	A*0201, Cw*0801	Japan	PCR-SSP	432	HAM	Protective	[36]
	HLA-A*02 and Cw*08	Peru	PCR-SSP	165	HAM	No	[37]
	HLA-G-14-bp deletion	Brazil	PCR	150	HAM ATLL	No	[38]
	B*07, DRB1*01:01	Spain	Bead array	40	HAM	Susceptibility	[39]
	A*02, A*24, Cw*08	Iran	PCR-SSP	50	HAM	No	[40]
	HLA-G 3’-UTR14 bp ins/del	Brazil	PCR	235	HAM	Risk for infection	[41]
	HLA-G 3’-UTR14 bp del/del, +3003TT and +3142CC				HAM	Protective	
	HLA-G 3′-UTR +3142C, +3003CT and +3142CC				HAM	Susceptibility	
	A*02, A*24, Cw*08, and B*5401	Iran	PCR	71	HAM	No	[42]
	HLA-C	Brazil	qPCR	241	HAM	No	[43]
	HLA-B*54	Japan	Mathematical modeling	392	HAM	Detrimental effect	[44]
	HLA-A*02:07, HLA-B*57 and HLA-C*08				HAM	Protective	
**KIR**	KIR3DS1	Peru	PCR-SSP	165	HAM	No	[37]
	KIR2DL2	Japan	PCR-SSP	402	HAM	Protective	[45]
	KIR3DS1	Jamaica	PCR-SSP	111	HAM ATLL	No	[46]
		Brazil	PCR-SSP	216	HAM	No	
		Japan	PCR-SSP	402	HAM	No	
	KIR2DL2	Japan	Mathematical modeling	392	HAM	Protective	[44]
	KIR3DL1				HAM	Detrimental effect	
	KIR2DL2, KIR2DL3, KIR2DS2 and KIR2DS3	Brazil	qPCR	246	HAM	No	[43]

PCR (Polymerase chain reaction); PCR-RFLP (Polymerase chain reaction—Restriction fragment length polymorphism); SSOPs (Sequence-specific oligonucleotide probes); PCR-SSP (Polymerase chain reaction—sequence-specific primers); ARMS (Amplification refractory mutation system); Ins (Insertion); Del (Deletion); N (Sample size).

**Table 2:** Main polymorphisms in cytokine, NF-kB1, CYP1A1, MBL, and apoptotic genes associated with HTLV-1 infection.

Gene	Alleles/Genotype	Country	Method	N	Disease	Association	Reference
**LT-α**	LT-α*A/G	Japan	PCR-RFLP	261	HAM	Susceptibility	[30]
					ATLL	No	
**TNFR2**	TNFR2* 196R	Japan	PCR-SSP	261	HAM	Susceptibility	[30]
					ATLL	No	
**IL1-β**	−511*1/2	Japan	PCR-RFLP	368	HAM	No	[52]
**IL-17A**	−197*G/A	Brazil	qPCR	116	HAM	No	[53]
**IFN-γ**	+874*A/T	Brazil	qPCR	453	HAM	Risk	[54]
	+874*A/T	Brazil	qPCR	148	HAM	No	[55]
	+874*A/T	Brazil	PCR-SSP	700	HAM	No	[56]
**IL-18**	−607*AC	Brazil	qPCR	148	HAM	Risk	[55]
	−607*CC					Protective	
	−137*C/G					No	
**TNF-α**	−1031*C (T > C)	Japan	PCR-SSOPs	652	Uveitis	Risk	[29]
	−863*A (C > A)					Risk	
	−1031*T > C and −863*C/A	Japan	PCR-SSP	261	HAM	No	[30]
	−857*T (C/T)				HAM	Susceptibility	
					ATLL	No	
	−857*T (C/T)	Japan	PCR-SSOPs	151	ATLL	Risk	[57]
	−1031TT (T/C)	Japan	qPCR	2180	Atherosclerosis	Risk	[58]
	−380*G/A	Brazil	PCR-SSP	700	HAM	No	[56]
**TGF-β**	+10*T/C and +25*G/C	Brazil	PCR-SSP	700	HAM	No	[56]
**IL-10**	−592*A (C/A)	Japan	PCR-RFLP	368	HAM	No	[52]
	−592*A (C/A)	Japan	PCR-RFLP	535	HAM	Low risk	[59]
	−3575(T/A), −2849 (G/A), −2763 (C/A), −1082 (A/G) and −819 (T/C)					No	
	−592*A (C/A)	Brazil	PCR-RFLP qPCR	233	HAM	No	[60]
	−1082*G/A, −819*C/T and −592*C/A (Haplotype “ATA”)	Iran	ARMS-PCR	332	HAM	Susceptibility	[61]
	819*T/C	Japan	qPCR	2180	Atherosclerosis	No	[58]
	−1082*A/G, −592C/A and −819C/T	Brazil	PCR-SSP	700	HAM	No	[56]
	+4976*A/G, −819*A/G	Brazil	qPCR	678	HAM	No	[62]
	−1082*A (A/G)					Protective	
**IL-6**	−634*G/C	Japan	PCR-RFLP	368	HAM	Susceptibility	[52]
	−634*G/C	Brazil	PCR-RFLP qPCR	233	HAM	Susceptibility	[60]
	−174*G/C, −572*G/C and −597*G/A					No	
	−174*G/C	Brazil	PCR-SSP	700	HAM	No	[56]
**IL-28B**	rs8099917 *T/G	Japan	PCR and Sequencing	340	ATLL	No	[63]
	rs12979860 *C/T	Spain	qPCR	199	HAM	Risk	[64]
	rs12979860 *C/T	Brazil	PCR and Sequencing	112	HAM	No	[65]
	rs8099917 *GG (T > G)	Brazil	qPCR	229	HAM	Risk	[66]
	rs12979860 *CT (C > T)					Risk	
	rs8099917 T/G, rs12979860 C/T and rs8103142 T/C Haplotypes TTG and CTT	Brazil	pPCR		Arthropathy	Susceptibility for infection	[67]
	Haplotype TTG					Risk	
**Fas/FasL**	−670*AA (A > G)	Brazil	PCR-RFLP	134	ATLL	Severity	[68]
	−670*GG (A > G)					Survival	
	−670*AA (A > G)	Brazil	PCR-RFLP		HAM	Risk	[69]
	−670*GG (A > G)					Susceptibility for infection	
	−670*AA (A/G)	Peru	PCR Kbiosciences	216	HAM	Risk	[70]
	−844*C/T and −1377*G/A					No	
	−670*A > G	Brazil	PCR-RFLP	25	ATLL	↑Apoptosis	[71]
**MBL2**	Mbl*B	Japan	PCR-RFLP	143	Asymptomatic	↓PV load	[72]
	Mbl*A and Mbl*D	Brazil	PCR-RFLP	182	Asymptomatic	No	[73]
	Mbl*B					↑PV load	
	−550*H/L and −221*X/Y LYLX genotype	Brazil	PCR-SSP	174	Asymptomatic	Risk for infection	[74]
**NF-kB1**	94ATTG ins/del	Japan	qPCR	2180		No	[58]
**GSTM1**	+/−	Japan	PCR-SSOPs	151	ATLL	No	[57]
**GSTT1**	+/−						
**CYP1A1**	+/−						
**BCL2**	+/−						

PCR (Polymerase chain reaction); PCR-RFLP (Polymerase chain reaction-Restriction fragment length polymorphism); SSOPs (Sequence-specific oligonucleotide probes); PCR-SSP (Polymerase chain reaction - sequence-specific primers); PV (Proviral load); ↑ Increase; ↓ Reduction. Ins (Insertion); Del (Deletion); +/− (Presence/Absence); N (Sample size).
